# Ribonuclease A Family Member 2 Promotes the Malignant Progression of Glioma Through the PI3K/Akt Signaling Pathway

**DOI:** 10.3389/fonc.2022.921083

**Published:** 2022-06-07

**Authors:** Tingfeng Wu, Yongxiu Chen, Liying Yang, Xiangyu Wang, Ke'en Chen, Dianshuang Xu

**Affiliations:** ^1^ Department of Neurosurgery, The First Affiliated Hospital of Jinan University, Guangzhou, China; ^2^ Gynecology Department, Guangdong Women and Children Hospital, Guangzhou, China; ^3^ Institute of Pathology, Tongji Hospital, Tongji Medical College, Huazhong University of Science and Technology, Wuhan, China

**Keywords:** glioma, RNASE2, PI3K/AKT, overexpression, knockdown

## Abstract

The treatment of patients with glioma still faces many difficulties. To further optimize treatment, it is necessary to identify more accurate markers as treatment targets and predict prognostic indicators. *RNASE2* was identified as a differentially expressed gene (DEG) in glioma tissues using bioinformatics analysis. In glioma microarrays, 31.21% (54/173) and 68.79% (119/173) patients showed low and high RNASE2 protein expression levels, respectively. RNASE2 protein levels were considerably correlated with age, WHO grade, relapse, and death. Both mRNA and protein levels were associated with the overall survival of patients with glioma. To investigate the role of RNASE2, it was overexpressed or silenced in glioma cells. RNASE2 overexpression promoted cell proliferation, migration, and invasion. In addition, its overexpression promoted the growth of subcutaneous tumors and lung metastasis of glioma cells. Key protein levels in the PI3K/Akt signaling pathway were upregulated by RNASE2 overexpression. In contrast, RNASE2 knockdown had the opposite effects. Furthermore, LY294002 blocked the effects of RNASE2 on the cell function of glioma cells. In conclusion, RNASE2 is a novel marker associated with the diagnosis and prognosis of patients with glioma, and it promotes the malignant progression of gliomas through the PI3K/Akt signaling pathway.

## Introduction

Glioma is a tumor derived from the neuroepithelium and the most common primary intracranial tumor (1). Traditional treatment strategies for glioma, including surgery, chemotherapy, and radiotherapy, have encountered a bottleneck in improving patient prognosis (2). Immunotherapy has brought new hope for patients with malignant tumors; however, the immunotherapy of glioma still faces many difficulties. Researchers have made many advances in understanding the biology of gliomas. Many molecular markers, such as 1p/19q co-deletion, MGMT methylation, and EGFR amplification, have been identified and widely recognized (3). In-depth research on these targets has benefited the molecular-targeted therapy of patients with glioma (4, 5). To further optimize the treatment of patients with glioma, however, it is necessary to identify more accurate markers as treatment targets and predict prognostic indicators.

Ribonuclease A family member 2 (RNASE2) is an RNA-binding protein that belongs to the pancreatic ribonuclease family. RNASE2 exhibits antiviral activity (6). Moreover, it was identified as a potential biomarker in idiopathic pulmonary arterial hypertension (7). RNASE2 mRNA is elevated in peripheral blood mononuclear cells from patients with systemic lupus erythematosus, and RNASE2 silencing suppresses the production of age-associated B cell subsets *in vitro* (8). Furthermore, it is differentially expressed and related to prognosis in gastric cancer and clear cell renal cell carcinoma (9, 10). RNASE2 expression is also significantly higher in colorectal cancer stem cells (CD133^+^) than in CD133^−^ colorectal cancer cells (11). Proteomic analysis of urine showed that RNASE2 levels are higher in the urine of patients with prostate cancer than in that of patients with bladder cancer or benign prostate hyperplasia (12). At present, there are no reports on the function of RNASE2 in cancer.

PI3K/Akt signaling plays a pivotal role in the occurrence of cancers (13, 14). The aberrant activation of this pathway is associated with poor prognosis in patients with glioma (15, 16). Many functional proteins and non-coding RNAs regulate the PI3K/Akt signaling pathway (17–19). Therefore, inhibitors targeting the PI3K/Akt pathway have emerged as potential treatments for glioma (15, 20).

In this study, we aimed to analyze the expression pattern and role of RNASE2 in glioma. Moreover, we hypothesized that RNASE2 might play its role in the tumorigenesis of glioma by activating PI3K/Akt signaling. Our findings will further enrich our understanding of the biology of glioma and offer a new theoretical basis for the development of new glioma inhibitors.

## Materials and Methods

### mRNA Expression Profile Analysis in the Gene Expression Omnibus Database

Two datasets (GSE4290 and GSE50161) were collected from GEO, a public functional genomics data repository. For the GSE4290 dataset, RNA expression data were collected from the brain tissues of 77 patients with glioblastoma and 23 patients with epilepsy (used as non-tumor samples). For the GSE50161 dataset, RNA expression data were collected from 117 human glioma tissues (glioblastoma, ependymoma, and medulloblastoma) and 13 human normal brain tissues. The Limma and edgeR packages were used to identify genes with statistical differences (both *p* <  0.05 and log [fold change] > 2). An overall survival analysis of the differentially expressed genes (DEGs) in both the GSE4290 and GSE50161 datasets was performed using The Cancer Genome Atlas (TCGA) database. The DEGs associated with overall survival in both the GSE4290 and GSE50161 datasets were used for subsequent analyses.

### RNASE2 Protein Level Analysis in Glioma Tissue Microarrays

A glioma tissue microarray was purchased from Shanghai Outdo Biotech Company (serial number: HBraG180Su01; Shanghai, China). Immunohistochemical analysis of RNASE2 on the microarray was performed by the Shanghai Outdo Biotech Company. A primary antibody against RNASE2 was purchased from Abnova Corporation (Taibei, China) and diluted at a ratio of 1:100. The staining intensity and staining extent of RNASE2 protein were assessed by professional pathologists (21).

### RNASE2 Overexpression and Knock Down

To construct an RNASE2-overexpressing lentiviral plasmid, the coding sequence of RNASE2 was obtained through PCR amplification using the primers (5’-3’) ccggaattcgccaccATGGTTCCAAAACTGTTCACTTCCCAAATTTG and ataagaatgcggccgcTTAGATGATTCTATCCAGGTGAACTGGAAC. PCR products and pLVX-mCMV-ZsGreen-puro plasmids were digested using *Eco*RI and *Not*I. The digested fragments were ligated using T4 ligase, yielding a recombinant plasmid named pLVX-RNASE2. To construct an RNASE2-silencing lentiviral plasmid, the sequence 5’-tatgacctgtcctagtaacaattcaagagattgttactaggacaggtcatatttttt-3’ was cloned into the plasmid pLent-U6-GFP-Puro *via* artificial DNA synthesis. The recombinant plasmid was then named pLent-shRNA-RNASE2. Both the pLVX-RNASE2 and pLent-shRNA-RNASE2 plasmids were used for packaging lentiviruses, named LV-ovRNASE2 and LV-shRNASE2, respectively. Empty plasmids were used to package the negative control lentiviruses, named LV-ovNC and LV-shNC. U87 MG and U251 MG cells were infected with LV-ovNC, LV-ovRNASE2, LV-shNC, and LV-shRNASE2 to obtain the groups named ovNC, ovRNASE2, shNC, and shRNASE2, respectively. Infected cells were screened using puromycin treatment, and positively screened cell lines were used for cell function analysis.

### Cell Function Analysis

To analyze the effect of RNASE2, the cell proliferation rate, level of apoptosis, migration, and invasion capabilities of cells in the shNC, shRNASE2, ovNC, and ovRNASE2 groups were measured. After culturing for 1, 2, 3, and 4 days, the optical density of cells at 450 nm (OD_450 nm_) was measured according to the method of the Cell Counting Kit-8 (KeyGen, Nanjing, China). The cell proliferation rate was calculated using the OD_450 nm_ in the following equation: Cell proliferation rate = (OD value at other time points/OD value at 0 h − 1) × 100% (same sample). After culturing for 48 h, the level of apoptotic cells was determined using the Annexin V-APC/7-AAD Apoptosis Kit (Abnova Corporation, Taibei, China). Cell migration and invasion were evaluated using Transwell and Transwell-Matrigel assays (22).

To investigate whether RNASE2 plays a role through the PI3K/AKT pathway, LY294002, a PI3K inhibitor, was used to treat cells in the ovRNASE2 group, termed ovRNASE2+LY294002. The ovNC and ovRNASE2 groups were treated with DMSO for use as controls and were named ovNC+DMSO and ovRNASE2+DMSO, respectively. The cell proliferation rate, level of apoptosis, and migration and invasion capabilities of these groups were measured using the same methods as those described above.

### Western Blotting

Total proteins from the shNC, shRNASE2, ovNC, and ovRNASE2 groups were isolated. Protein concentration quantitation, sodium dodecyl sulfate-polyacrylamide gel electrophoresis, and western blotting assay were performed as described by Guo and Xia (22). The primary antibodies against RNASE2, PI3K p85, total AKT1, phosphorylated AKT1, and GAPDH used in this study were purchased from Abnova Corporation.

### Animal Experiments

To analyze the *in vivo* effect of RNASE2 on tumor growth, 40 BALB/c athymic nude mice were divided into eight groups (n = 5 per group). The eight groups of mice were subcutaneously injected with cells from the shNC, shRNASE2, ovNC, or ovRNASE2 group (5 × 10^6^ cells/mouse). Tumor width (W) and length (L) were measured using calipers at 6, 12, 17, 23, and 26 days after injection. The formula (L × W^2^)/2 was used to calculate the tumor volume.

To analyze the *in vivo* effect of RNASE2 on lung metastasis, 32 athymic nude mice were divided into eight groups (n = 4 per group). The eight groups of mice were injected intravenously with cells from the shNC, shRNASE2, ovNC, or ovRNASE2 group (5 × 10^5^ cells/mouse) resuspended in 100 μl of Hank’s balanced salt solution. After 6 weeks, the mice were euthanized and their lung tissues were isolated. After fixing with 4% paraformaldehyde, lung tissues were embedded using paraffin. Hematoxylin and eosin staining were performed to identify tumor loci in lung tissues.

### Label-Free Quantitative Proteomics

U87 MG cells of the shNC, shRNASE2, ovNC, and ovRNASE2 groups were harvested for label-free quantitative proteomics analysis, which was performed by Fitgene Biotech Co., Ltd. (Guangzhou, China). Peptides (number ≥2) were used to identify the differentially expressed proteins. Peptide ratios of shRNASE2 versus shNC or ovRNASE2 versus ovNC were calculated. The expression of proteins with a ratio ≥1.2 was considered upregulated and that of proteins with a ratio ≤0.833 was considered downregulated. Proteins with downregulated expression in shRNASE2 and upregulated expression in ovRNASE2, and those with upregulated expression in shRNASE2 and downregulated expression in ovRNASE2, were used for KEGG analysis to analyze the pathways involved.

### Statistical Analysis

Statistical analysis of the differences between shNC and shRNASE2 or between ovNC and ovRNASE2 was performed using an unpaired t-test. Statistical analysis of the differences between ovNC+DMSO, ovRNASE2+DMSO, and ovRNASE2+LY294002 was performed using one-way analysis of variance. All the data in the bar graphs are expressed as the mean ± standard deviation. The significance of the correlation between RNASE2 protein levels and clinicopathological features of patients with glioma was determined using a χ^2^ test. A log-rank (Mantel-Cox) test was used to assess the correlation between RNASE2 protein levels and overall survival, or RNASE2 protein levels and disease-free survival.

## Results

### Gene Profiling in Glioma Tissues

To identify DEGs in glioma tissues, we queried the GEO data repository and downloaded two RNA expression datasets from glioma samples (GSE4290 and GSE50161). As shown in **Supplementary File 1**, 702 DEGs were identified, including 415 downregulated and 287 upregulated genes. Subsequently, we analyzed the association between these DEGs and the overall survival of patients with glioblastoma using TCGA. The gene symbols and gene IDs of the DEGs associated with overall survival are shown in **Table 1**.

**Table 1:** DEGs associated with overall survival in both the GSE4290 and GSE50161 datasets.

Upregulation		
Gene symbol	Gene ID	*p* value
AEBP1	ENSG00000106624.8	0.0000309
HOXB2	ENSG00000173917.10	0.00082
HOXC6	ENSG00000197757.7	0.00119
NMB	ENSG00000197696.9	0.00141
TNFRSF11B	ENSG00000164761.8	0.00301
RNASE2	ENSG00000169385.2	0.00373
MSTN	ENSG00000138379.4	0.00486
P4HB	ENSG00000185624.14	0.00567
IGFBP4	ENSG00000141753.6	0.0062
TGFBI	ENSG00000120708.16	0.00631
FLNC	ENSG00000128591.15	0.00678
PCOLCE	ENSG00000106333.12	0.00708
SDC1	ENSG00000115884.10	0.00766
LY96	ENSG00000154589.6	0.00782
MTHFD2	ENSG00000065911.11	0.00884
**Downregulation**		
**Gene symbol**	**Gene ID**	***p* value**
PTPRN	ENSG00000054356.13	0.0000653
ITPKA	ENSG00000137825.10	0.00191
PLK2	ENSG00000145632.14	0.00193
GPC5	ENSG00000179399.13	0.00236
ADARB1	ENSG00000197381.15	0.00718
PTPRN2	ENSG00000155093.17	0.00754
RGS4	ENSG00000117152.13	0.00938
PTPRN	ENSG00000054356.13	0.0000653

### Expression Characteristics of RNASE2 in Glioma Tissues

**Figure 1:**
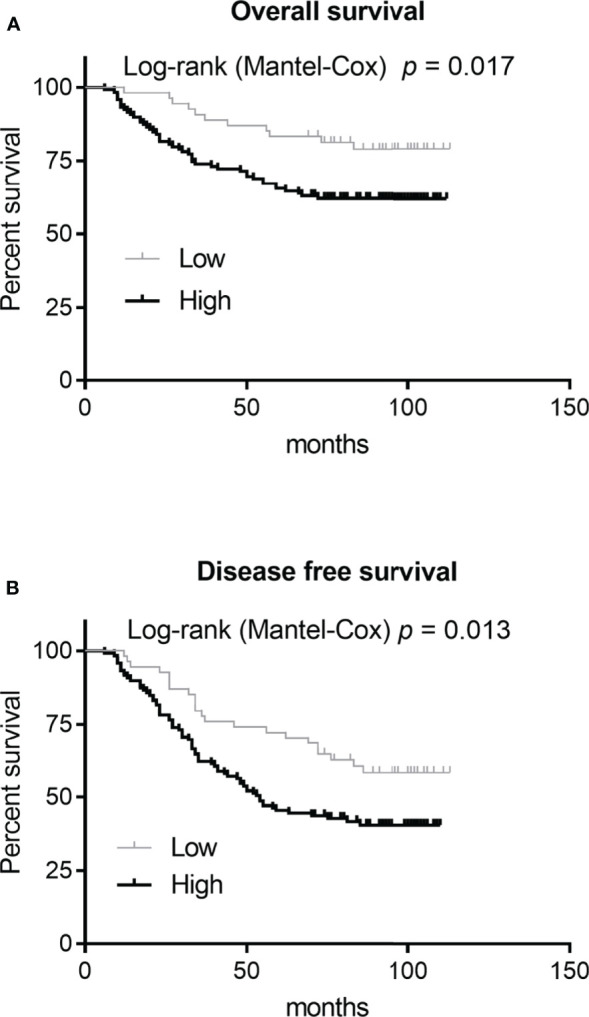
Correlation analysis between RNASE2 protein levels and patient prognosis. Based on the RNASE2 protein levels on the glioma tissue microarray, 173 patients with glioma were grouped into low (score <2) and high (score ≥2) expression groups. A Log-rank (Mantel-Cox) test was used to assess the correlation between RNASE2 levels and overall survival, or RNASE2 levels and disease-free survival.

After removing genes with known functions, we selected RNASE2 for subsequent expression characteristics analyses and functional studies. The total number of tissues evaluated in this analysis was 173. The RNASE2 protein was positively expressed in all the 173 (100%) glioma tissues. RNASE2 protein expression levels were also determined using the staining intensity score. According to the RNASE2 protein expression level, the 173 patients were divided into low (score <2) and high (score ≥2) expression groups. The low and high expression groups were composed of 54 (31.21%) and 119 (68.79%) patients, respectively. As shown in **Table 2**, RNASE2 protein levels were significantly correlated with age, WHO grade, relapse, and death. Moreover, patients in the low RNASE2 expression group had longer disease-free survival and overall survival than those in the high expression group (**Figure 1**).

**Table 2:** Correlation between RNASE2 levels and clinicopathological features of patients with glioma.

Characteristic	N = 173	RNASE2 protein expression		*p* value
		Low group (n = 54)	High group (n = 119)	
Sex				0.714
Male	106	32	74	
Female	67	22	45	
Age (years)		34.87 ± 17.62	44.05 ± 16.56	0.001
WHO grade				0.031
I+II	101	39	62	
III+IV	72	14	58	
Relapse				0.027
No	81	32	49	
Yes	92	22	70	
Death				0.023
No	117	43	74	
Yes	56	11	45	

### Regulation of Cell Proliferation by RNASE2

**Figure 2:**
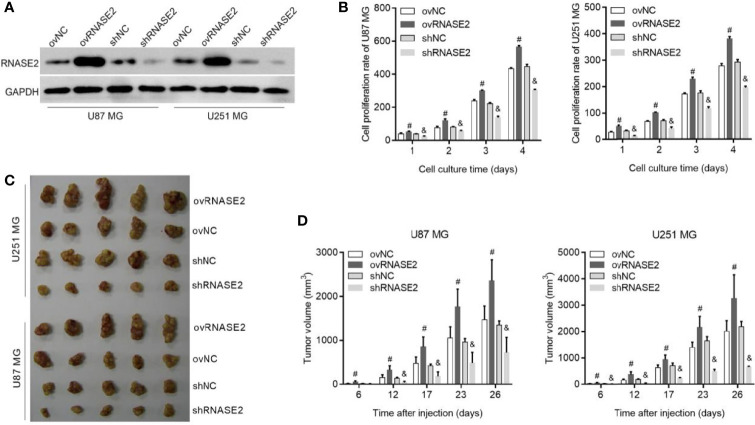
RNASE2 overexpression promotes the proliferation of U87 MG and U251 MG cells, while RNASE2 knockdown suppresses it. RNASE2-overexpressing (ovRNASE2) and RNASE2-silenced (shRNASE2) cells were constructed. The ovNC and shNC groups were used as control cells. **(A)** Western blot showing successful RNASE2 protein overexpression and knock down. **(B)** Cell proliferation was promoted by RNASE2 overexpression and suppressed by RNASE2 knockdown after culture for 1, 2, 3, and 4 days. **(C, D)** The growth of subcutaneous tumors was promoted by RNASE2 overexpression and suppressed by RNASE2 knockdown. Panel **(C)** shows a photograph of the subcutaneous tumors. Panel **(D)** shows the statistical results of tumor volume after cell injection for 6, 12, 17, 23, and 26 days. # indicates *p* < 0.05; ovRNASE2 versus ovNC. & indicates *p* < 0.05; shRNASE2 versus shNC.

RNASE2 was successfully overexpressed in ovRNASE2 cells and knocked down in shRNASE2 cells (**Figure 2A**). Next, we analyzed the effects of RNASE2 overexpression and knockdown on cell proliferation *in vitro* and *in vivo*. Compared to the control group, the ovRNASE2 and shRNASE2 groups showed higher and lower cell proliferation rates, respectively (**Figure 2B**). Similarly, the subcutaneous tumor volume was larger in the ovRNASE2 group and smaller in the shRNASE2 (**Figures 2C, D**). These results demonstrated that RNASE2 promotes glioma cell proliferation.

### Regulation of Glioma Cell Apoptosis by RNASE2

**Figure 3:**
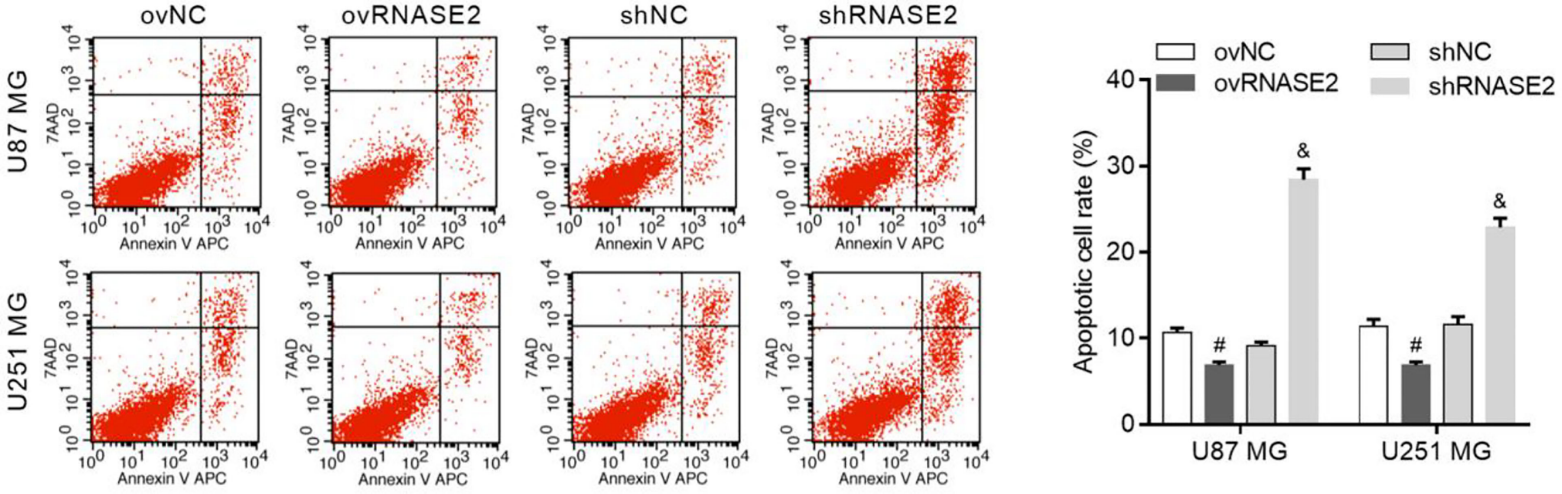
RNASE2 overexpression suppresses the apoptosis of U87 MG and U251 MG cells, while RNASE2 knockdown promotes it. RNASE2-overexpressing (ovRNASE2) and RNASE2-silenced (shRNASE2) cells were constructed. The ovNC and shNC groups were used as control cells. # indicates *p* < 0.05; ovRNASE2 versus ovNC. & indicates *p* < 0.05; shRNASE2 versus shNC.

Compared to the ovNC and shNC groups, the ovRNASE2 and shRNASE2 groups had lower and higher apoptotic cell rates, respectively (**Figure 3**). These results demonstrated that RNASE2 has a suppressive effect on glioma cell apoptosis.

### Regulation of Glioma Cell Migration and Invasion by RNASE2

**Figure 4:**
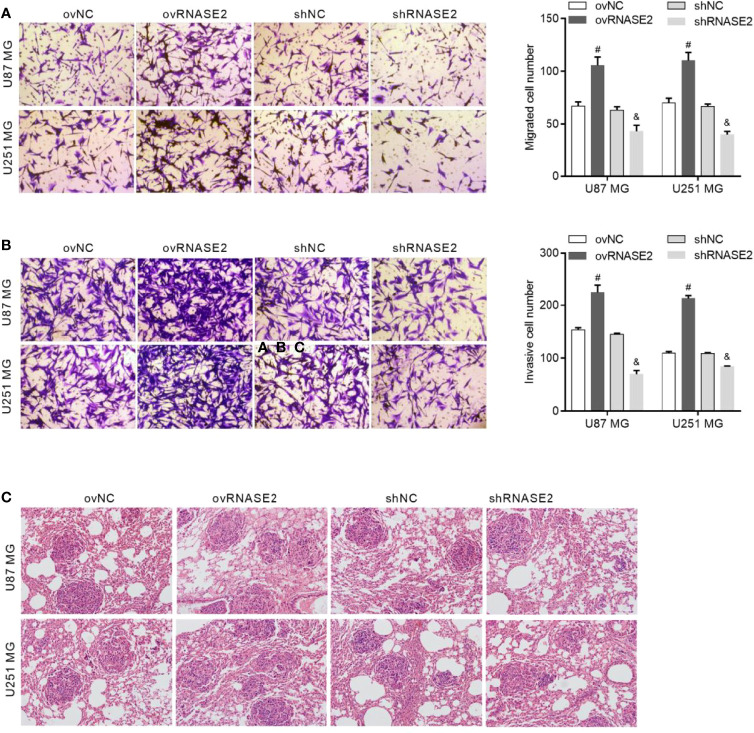
RNASE2 overexpression promotes the migration and invasion of U87 MG and U251 MG cells, while RNASE2 knockdown suppresses it. RNASE2-overexpressing (ovRNASE2) and RNASE2-silenced (shRNASE2) cells were constructed. The ovNC and shNC groups were used as control cells. **(A)** Transwell assay showing that RNASE2 overexpression increased the number of migrating cells, while RNASE2 knockdown reduced it. **(B)** Transwell-Matrigel assay showing that RNASE2 overexpression increased the number of invasive cells, while RNASE2 knockdown reduced it. **(C)** Lung metastasis model in nude mice showing that RNASE2 overexpression increased the number of tumor loci in lung tissue, and that RNASE2 knockdown reduced it. # indicates *p* < 0.05; ovRNASE2 versus ovNC. & indicates *p* < 0.05; shRNASE2 versus shNC.

Compared to the control cells, ovNC and shNC, the ovRNASE2 and shRNASE2 groups had a higher and lower number of migrated and invasive cells, respectively **Figures 4A, B**. Similarly, the number of tumor loci in lung tissue was higher and lower in the ovRNASE2 and shRNASE2 groups, respectively (**Figure 4C**). The above results demonstrated that RNASE2 promotes the migration and invasion of glioma cells.

### Identification of Differentially Expressed Proteins Regulated by RNASE2 in Glioma Cell Lines

To explore the underlying mechanism of RNASE2 in glioma, differentially expressed proteins in the shRNASE2 and ovRNASE2 groups of U251 MG cells were identified. **Supplementary File 2** shows the proteins upregulated by RNASE2 silencing and downregulated by RNASE2 overexpression. **Supplementary File 3** shows the proteins downregulated by RNASE2 silencing and upregulated by RNASE2 overexpression. Subsequently, pathway annotation was performed on these differentially expressed proteins, and the pathways involved were enriched. The results of pathway enrichment are shown in **Supplementary Files 2** and **3**.

### RNASE2 Plays a Role in the PI3K/Akt Signaling Pathway

**Figure 5:**
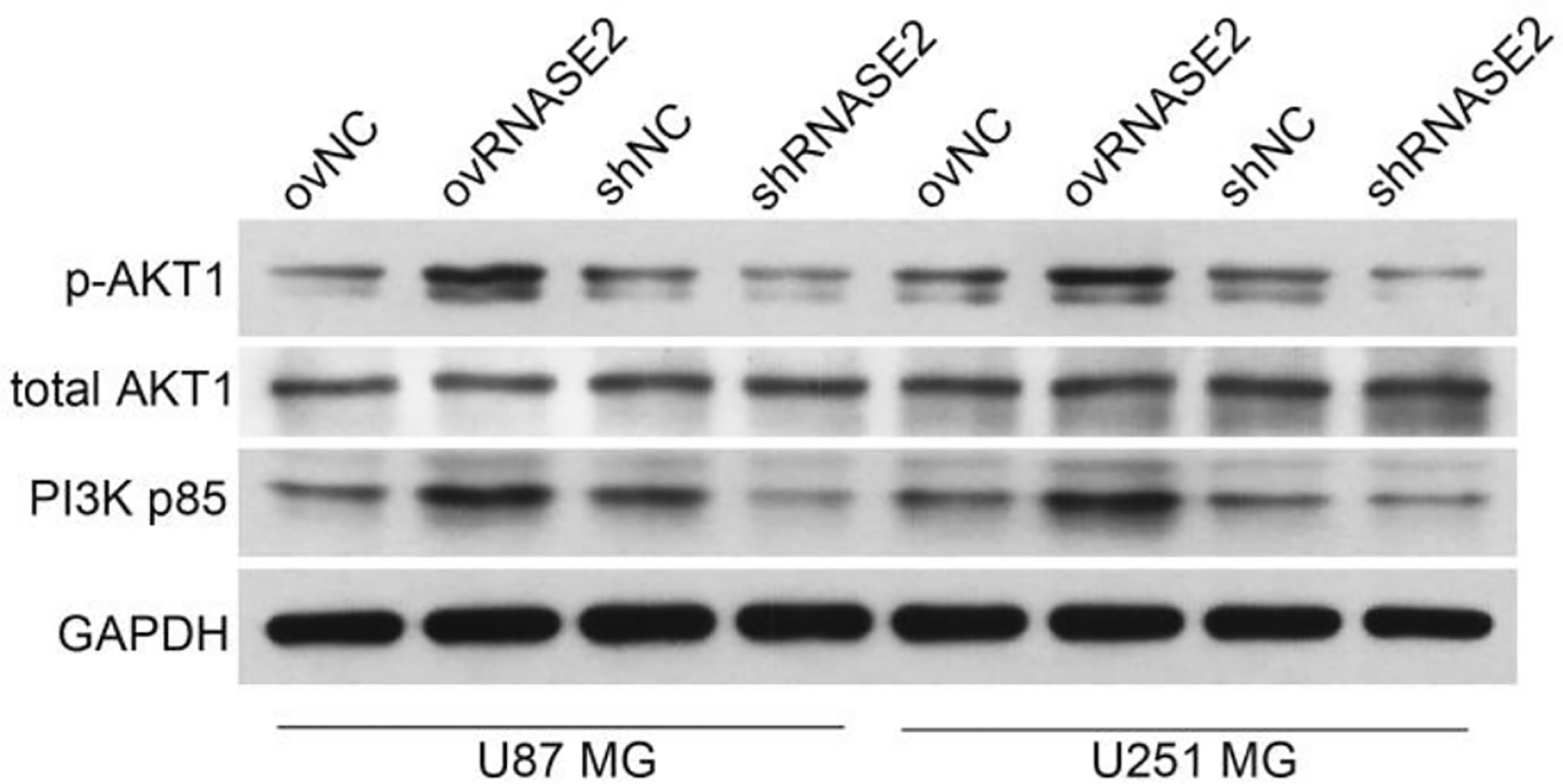
RNASE2 overexpression activates the PI3K/Akt signaling pathway, while RNASE2 knockdown inactivates it. RNASE2-overexpressing (ovRNASE2) and RNASE2-silenced (shRNASE2) cells were constructed. The ovNC or shNC groups were used as control cells. RNASE2 overexpression upregulated, while RNASE2 knockdown downregulated, the protein levels of p-AKT1 and PI3K p85. RNASE2 had no effect on total AKT1 levels.

Among all the enriched pathways, the PI3K/Akt signaling pathway attracted our attention because it is one of the main targets for drug design in glioblastoma (23). We then verified the role of RNASE2 in the PI3K/Akt signaling by measuring the protein expression of total AKT1, p-AKT1, and PI3K p85 in the ovNC, ovRNASE2, shNC, and shRNASE2 groups. Compared to the control cells, ovNC or shNC, the ovRNASE2 and shRNASE2 groups showed higher and lower protein levels of p-AKT1 and PI3K p85, respectively (**Figure 5**). RNASE2 had no effect on total AKT1 levels. The above results demonstrated that RNASE2 can activate PI3K/Akt signaling.

**Figure 6:**
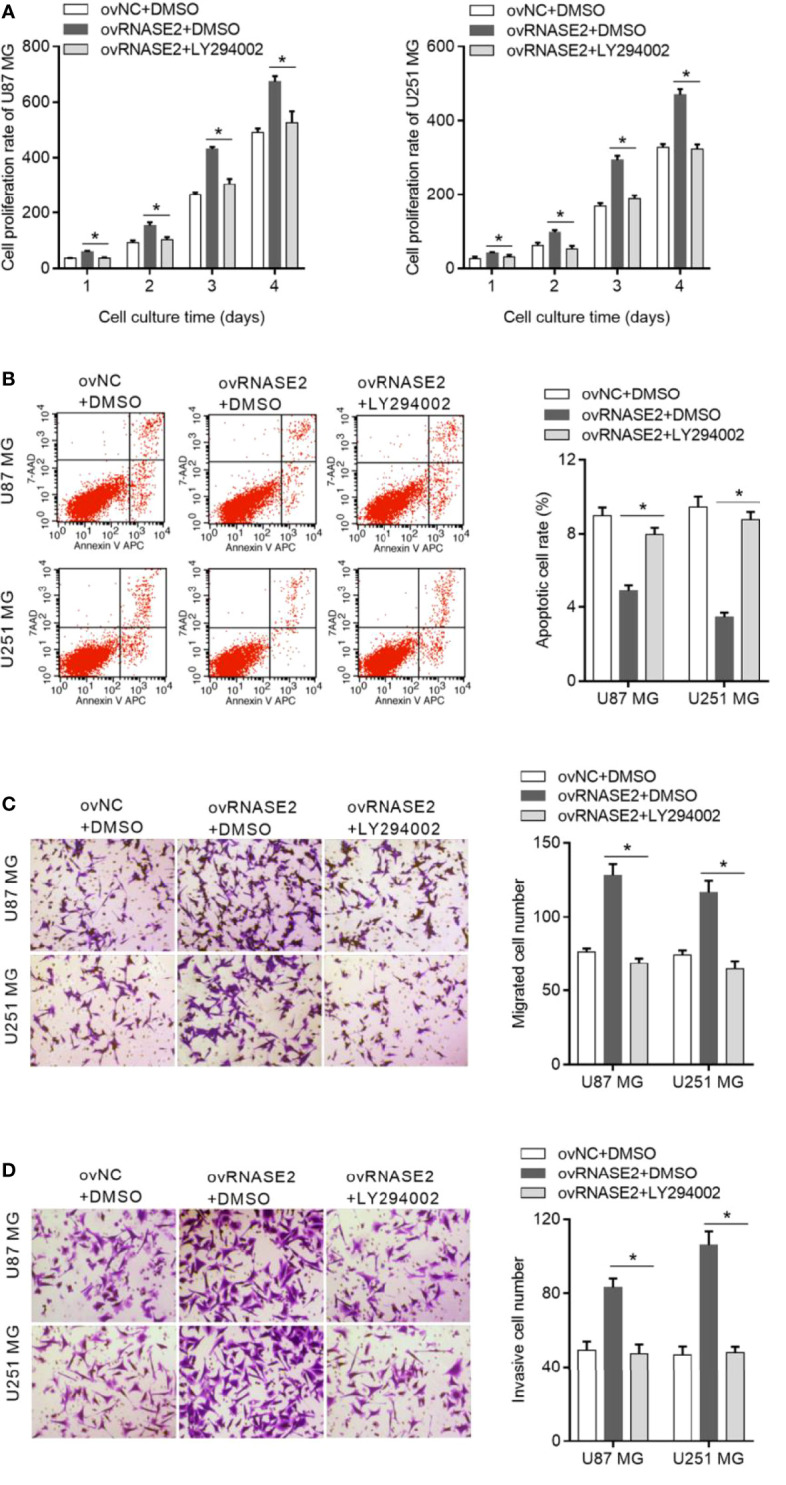
LY294002, a PI3K inhibitor, blocks the effect of RNASE2 overexpression on the function of U87 MG and U251 MG cells. LY294002 was used to treat cells overexpressing RNASE2 (ovRNASE2+LY294002). DMSO-treated cells were used as a control (ovNC+DMSO, ovRNASE2+DMSO). LY294002 alleviated the effect of RNASE2 overexpression on proliferation **(A)**, apoptosis **(B)**, migration **(C)**, and invasion **(D)**. * indicates *p* < 0.05; ovRNASE2+LY294002 versus ovRNASE2+DMSO.

To explore whether RNASE2 plays its role *via* the PI3K/Akt pathway, LY294002, a cell-permeable inhibitor of PI3K, was used to treat cells in the ovRNASE2 group. DMSO-treated cells were used as a control. Compared to the ovRNASE2+DMSO group, the ovRNASE2+LY294002 group had a lower cell proliferation rate (**Figure 6A**), higher apoptotic cell rate (**Figure 6B**), and lower number of migrated and invasive cells (**Figures 6C, D**). The above results showed that LY294002 can block the effects of RNASE2.

## Discussion

An abnormal expression of RNASE2 has been reported in colorectal cancer stem cells and bladder cancer tissues (11, 12); however, the function and mechanism of RNASE2 in cancer cells has not been studied. In this study, we investigated the role of RNASE2 in glioma using clinical samples, cell lines, and animal experiments. By analyzing two RNA expression datasets of glioma samples collected from the GEO data repository, we found that RNASE2 mRNA expression was upregulated in glioma samples and was associated with the overall survival of patients with glioma, suggesting that RNASE2 may play a regulatory role in glioma tumorigenesis. Similarly, a bioinformatics analysis from the TCGA database also indicated that RNASE2 mRNA expression is associated with the prognosis of patients with gastric cancer (9, 24). Next, we analyzed the expression characteristics of RNASE2 using glioma samples and explored its function by overexpressing and knocking down its expression in glioma cell lines.

The expression characteristics of proteins are essential for elucidating their functions. Therefore, we analyzed RNASE2 protein levels using glioma tissue microarrays. To the best of our knowledge, this is the first study examining RNASE2 protein expression in cancer tissues and its correlation with patient prognosis. We found that RNASE2 was positively expressed in all glioma tissues, and that the proportion of patients in the high expression group was 68.79%. These results indicate that RNASE2 is highly expressed in glioma tissues; however, no normal tissue was used for comparison in the analysis. This glioma tissue microarray has complete pathological information and follow-up information on the prognosis of all enrolled patients, which are important data for explaining the role of RNASE2. We found that RNASE2 protein levels were remarkably correlated with age, WHO grade, relapse, and death. RNASE2 had a higher expression rate in the glioma tissues of patients with advanced-stage disease than in those of patients with early disease. In addition, RNASE2 had a higher expression rate in glioma tissues collected from patients who had died or relapsed at the time of follow-up than in those collected from patients who survived or did not relapse at follow-up. These results indicate that RNASE2 can promote the occurrence and development of gliomas. This hypothesis is further supported by the finding that patients in the low RNASE2 expression group had longer disease-free survival and overall survival than those in the high expression group.

The expression characteristics of RNASE2 obtained in this study suggest that RNASE2 may play an oncogenic role in glioma. The promoting effect of RNASE2 overexpression on cell growth and metastasis further supports this hypothesis. Moreover, RNASE2 knockdown had a suppressive effect on cell growth and metastasis, indicating that inhibiting RNASE2 expression may be a strategy to suppress the genesis and development of glioma. Our results revealed that RNASE2 may be a potential molecular target for glioma drug development. To the best our knowledge, there have been no studies yet on the function of RNASE2 in other types of cancer cells; hence, this study reveals a novel role of RNASE2.

To further elucidate the mechanism of RNASE2, differentially expressed proteins regulated by RNASE2 were identified, and the pathways they are involved in were enriched. Among all the enriched pathways, PI3K/Akt signaling is the most reported signaling involved in tumorigenesis. The abnormal activation of the PI3K/Akt signal transduction pathway stimulates the malignant proliferation of cancer cells and enhances their invasion and metastatic ability (25, 26). We found that PI3K/Akt signaling could be activated by RNASE2 overexpression and inactivated by RNASE2 knockdown. Moreover, LY294002 blocked the effects of RNASE2 overexpression. These findings show that RNASE2 may play its role in glioma through its action on PI3K/Akt signaling. However, other pathways were also enriched. It must be acknowledged that RNASE2 may also act through multiple signaling pathways. This hypothesis will be explored in subsequent experiments.

## Conclusion

Taken together, our findings reveal that RNASE2 levels are related to the clinicopathological features of patients with glioma and that RNASE2 may play an oncogenic role by activating PI3K/Akt signaling. Hence, RNASE2 may be a novel molecular target for the development of targeted therapies against glioma.

## Data Availability Statement

The original contributions presented in the study are included in the article/**Supplementary Material**. Further inquiries can be directed to the corresponding author.

## Ethics Statement

The animal study was reviewed and approved by the Guangzhou Institutes of Biomedicine and Health, Chinese Academy of Sciences.

All experimental protocols involving animals were performed according to the institutional ethical guidelines for animal experiments at the Guangzhou Institutes of Biomedicine and Health, Chinese Academy of Sciences.

## Author Contributions

Conceptualization, TW and YC; methodology, TW, YC, LY, DX and KC; investigation, LY, DX, and KC; writing—original draft preparation, TW and YC; writing—review and editing, TW and YC; visualization, TW, YC and LY; project administration, TW and YC; funding acquisition, TW. All authors have read and agreed to the published version of the manuscript.

## Funding

This work was supported by grants from the Fundamental Research Funds for the Central Universities (Grant/Award Number: 21619346).

## Conflict of Interest

The authors declare that the research was conducted in the absence of any commercial or financial relationships that could be construed as a potential conflict of interest.

## Publisher’s Note

All claims expressed in this article are solely those of the authors and do not necessarily represent those of their affiliated organizations, or those of the publisher, the editors and the reviewers. Any product that may be evaluated in this article, or claim that may be made by its manufacturer, is not guaranteed or endorsed by the publisher.

## Supplementary Material

Supplementary File 1 DEGs in the GSE4290 and GSE50161 datasets.

Supplementary File 2 Identification and annotation of the top 20 pathways of proteins upregulated by RNASE2 silencing and downregulated by RNASE2 overexpression.

Supplementary File 3 Identification and annotation of the top 20 pathways of proteins downregulated by RNASE2 silencing and upregulated by RNASE2 overexpression.

## Abbreviations

DEG, differentially expressed gene; RNASE2, ribonuclease A family member 2; ovRNASE2, overexpressing RNASE2; shRNASE2, shRNA targeting RNASE2; TCGA, the Cancer Genome Atlas; PI3K/Akt, phosphatidylinositol 3-kinase/Protein kinase B; GEO, Gene Expression Omnibus; OD_450 nm_, optical density at 450 nm.
