# Supplementary material for: Ribonuclease A Family Member 2 Promotes the Malignant Progression of Glioma Through the PI3K/Akt Signaling Pathway
Source: Front Oncol. 2022 Jun 7;12:921083. doi: 10.3389/fonc.2022.921083 (PMC9211777; doi:10.3389/fonc.2022.921083)
Supplement: Supplementary File 1 — DEGs in the GSE4290 and GSE50161 datasets. [file DataSheet_1.docx]

**Differentially expressed genes both in GSE4290 and GSE50161 datasets**

1. **Up-regulated genes**


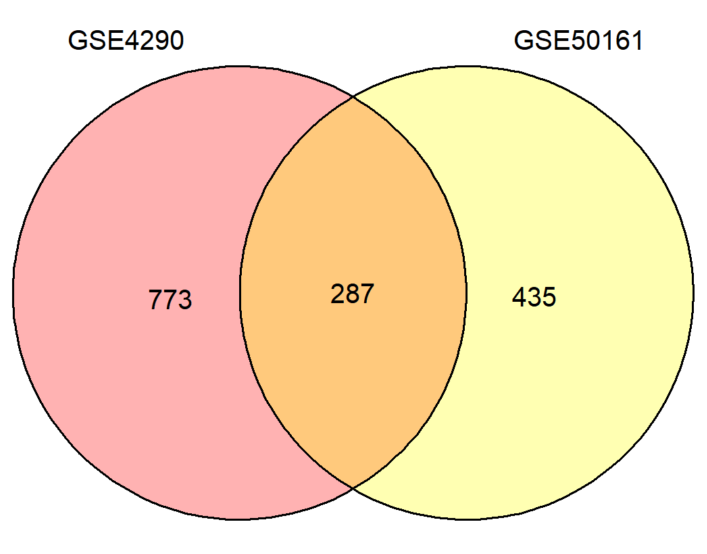


[1] "TOP2A" "NDC80" "IGFBP2" "SERPINH1" "MELK" "CNGA3"

[7] "FCGBP" "FOXM1" "ABCC3" "RRM2" "SERPINA3" "ID3"

[13] "HSPG2" "CCNB2" "TIMP4" "ANXA1" "EZH2" "TGFBI"

[19] "PTX3" "DLGAP5" "ZIC1" "C7orf57" "PLP2" "FOXD1"

[25] "COL1A2" "EMILIN1" "EMP3" "TNC" "CA3" "COL1A1"

[31] "PLA2G2A" "VMP1" "TTK" "CDK1" "TIMP1" "GBP1"

[37] "IGF2BP3" "LMNB1" "BARD1" "AGAP2-AS1" "IQGAP2" "ANXA2"

[43] "SOX11" "CDC20" "CFI" "KIF14" "F2R" "CSRP2"

[49] "PCOLCE" "PLAU" "S100A10" "CDC45" "AEBP1" "S100A11"

[55] "MTHFD2" "NMB" "CTSC" "SERPINE1" "SLC4A2" "KIF23"

[61] "TAGLN2" "PROS1" "CD151" "WWTR1" "GBP2" "CDK4"

[67] "RAD51AP1" "CD99" "CPVL" "SOX4" "EMP1" "NID1"

[73] "CENPF" "CDKN2C" "TP53" "MYBL2" "CENPE" "BUB1B"

[79] "KIAA0101" "ENPEP" "CHEK1" "PLTP" "ABCA1" "RHOC"

[85] "MYC" "KIAA0040" "SMC4" "VIM" "SRPX2" "PPIC"

[91] "HMMR" "VAMP8" "ITGA5" "RAB13" "PYGL" "MGP"

[97] "CSPG4" "HOXC6" "EGFEM1P" "SEC61G" "LCAT" "TGIF1"

[103] "TNFRSF1A" "ANXA5" "SLC16A4" "DYNLT1" "LAMC1" "CDK2"

[109] "LUM" "ZNF217" "PRDX4" "IGFBP7" "GAS1" "ACTL6A"

[115] "HOXA4" "S100A4" "PLSCR1" "CD93" "CRIP1" "LOXL2"

[121] "UBE2C" "MSN" "KIF11" "HLA-DRB1" "APOBEC3B" "IFI16"

[127] "DBF4" "HOXB2" "MCM2" "PSMB9" "BIRC5" "IGFBP4"

[133] "SERPING1" "ESM1" "KNTC1" "SOCS2" "NUDT1" "CSTA"

[139] "NMI" "MMP9" "ZFP36L2" "HMOX1" "RFX2" "IL1RAP"

[145] "SPARC" "MMP2" "TYMS" "DDX39A" "COL6A3" "TK1"

[151] "CAPG" "P3H4" "MYOF" "WNT5A" "LGALS3BP" "PCNA"

[157] "RBP1" "AURKA" "HCP5" "LY96" "PLOD2" "PRSS23"

[163] "SFRP4" "LOXL1" "EDNRA" "VCAN" "ADGRE5" "STK17A"

[169] "P4HB" "CD58" "CNN3" "RCN1" "ECM2" "SCIN"

[175] "MDFI" "HLA-DPB1" "CYBA" "CALCRL" "ELAVL1" "RNASE2"

[181] "TNFRSF11B" "RPE65" "SP100" "LRRN4CL" "PTK7" "HK2"

[187] "RP2" "OAS1" "SLPI" "RBBP8" "VCAM1" "POLD1"

[193] "SNRPG" "IGFBP7-AS1" "CDC25A" "DPYSL3" "MAD2L1" "MCM3"

[199] "RFXANK" "CLIC4" "KDELR2" "ITGB2" "ADAM9" "ODC1"

[205] "IQUB" "ABCB4" "RAD51" "ZBTB20" "HSD17B10" "HMGN1"

[211] "HOXD4" "NID2" "ZNF124" "RNASEH2A" "SNAPC1" "GUSB"

[217] "FOXJ1" "ZYX" "SDC1" "IGFBP1" "IQGAP1" "ANPEP"

[223] "MSTN" "PPIB" "LPCAT1" "GNG5" "DAP" "PSPH"

[229] "IFI44L" "MEST" "STC2" "CXCL10" "TSPO" "GNAI3"

[235] "ST6GALNAC2" "CEP135" "RCC1" "BRCA1" "LSM7" "HAT1"

[241] "LRP10" "CDH11" "RUVBL1" "LBR" "CTSS" "LIF"

[247] "DPYD" "EFEMP2" "MVP" "EXO1" "PTTG1" "MTMR11"

[253] "CKAP4" "PRKX" "TLR3" "CD63" "ACOX2" "SSR4"

[259] "SLC7A7" "COL5A1" "PBX3" "SEC11A" "RBL1" "HNMT"

[265] "TIMELESS" "CHSY1" "TGFB1" "ZNF107" "FLNC" "ZKSCAN7"

[271] "LOC100131541" "RAB42" "FAS" "BCL2A1" "DTYMK" "ZNF621"

[277] "BMP2" "ACTA2" "ZWINT" "VAMP5" "HOXC4" "LRRC17"

[283] "TRAM1" "TRAF4" "ST5" "LAS1L" "ZNF516"

1. **Down-regulated genes**


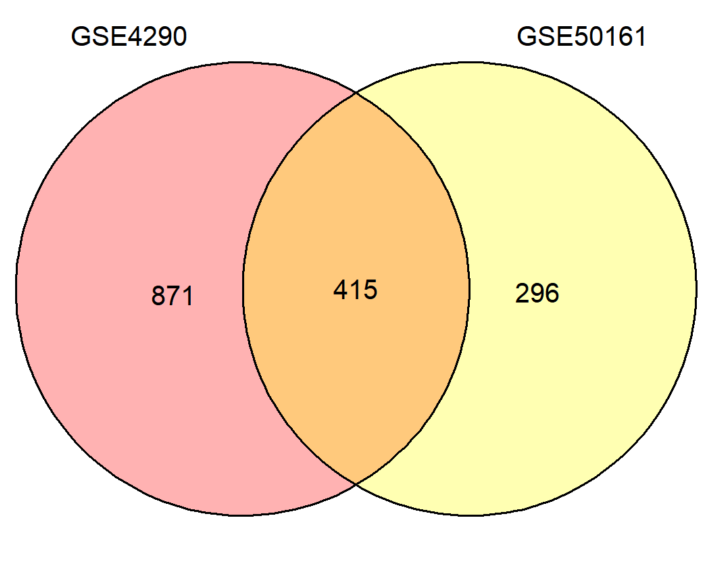


[1] "AKAP11" "GNB5" "ENTPD6" "FAM50B" "GNAZ" "RAPGEF2"

[7] "KRTAP5-AS1" "GABRA6" "AP3M2" "SOHLH1" "MAPK9" "ANK2"

[13] "FAM131B" "GSTM5" "PIP5K1B" "PPP1R12B" "ETS2" "LMTK3"

[19] "RFPL2" "HSPH1" "MAN2A2" "ZNF702P" "GAS7" "MAFG"

[25] "RAB11FIP2" "ACTN2" "RABGAP1L" "PRPH2" "LRRTM2" "C2CD2L"

[31] "PIN1" "DRP2" "PIK3C2B" "FGFR2" "RALGPS1" "DLGAP1"

[37] "OXCT1" "GJC2" "ADGRB2" "CLSTN1" "SSX2IP" "NEBL"

[43] "ADARB1" "CLUL1" "TMEM266" "MAP3K10" "FGFR3" "USP46"

[49] "ABCG4" "RNASE1" "ATP6V1B2" "UROS" "RASGRP1" "INPP5A"

[55] "MAPRE2" "C1orf204" "CCDC85B" "PYGM" "NAGPA" "RGS11"

[61] "NPTN" "MYCBP2" "NDEL1" "SATB1" "INHBA-AS1" "TNFSF9"

[67] "PLD3" "SIAH3" "PPP3CA" "OAT" "NR3C2" "PALM"

[73] "CTNNA2" "CDK5" "SH3BP5" "CADPS" "SNAI3-AS1" "ACTR3B"

[79] "ABCC12" "ARHGEF9" "TSPAN7" "PRRG1" "LY6E" "PTPRN2"

[85] "RNF41" "SLIT3" "SLC4A3" "LZTS3" "REEP1" "R3HDM1"

[91] "NEURL1" "PCDH8" "MAPK10" "PRKACB" "AKAP6" "DHCR24"

[97] "DLG4" "WDR47" "MAP2K4" "KCNN1" "UCHL1" "STAT4"

[103] "MBNL2" "ARHGAP44" "MMD" "GUCA1A" "STMN1" "PTK2B"

[109] "OSBPL1A" "LANCL1" "APBB1" "PDE4A" "KIF3C" "VAMP2"

[115] "ENO2" "TRHDE-AS1" "BAIAP2" "ATP1B1" "SCN1B" "MDH1"

[121] "LDOC1" "SLC31A2" "AGAP2" "NPAS4" "CLSTN3" "LPAR1"

[127] "ALDOC" "MCF2" "SLC9A6" "PDIA2" "RYR1" "PKP4"

[133] "SV2A" "KIAA0408" "SLC8A3" "LOC283484" "KCNG3" "MAP2K1"

[139] "NR4A2" "PPP3R1" "FKBP1B" "AGTPBP1" "B4GAT1" "CORO6"

[145] "OPTN" "MGAT4C" "CARTPT" "IQSEC1" "LOC283588" "GFOD1"

[151] "LOC375196" "ACOT7" "EGR4" "NBL1" "ELMO1" "PNOC"

[157] "TPD52L1" "ASPHD1" "EVI2A" "HPRT1" "RAP1GAP" "ENPP4"

[163] "LY86-AS1" "SLC6A1" "PRKAR2B" "FAAH" "SNPH" "KIT"

[169] "GLRB" "ADGRB3" "MAPRE3" "ME1" "RAB33A" "RIT2"

[175] "TSPOAP1" "S100A1" "PPEF1" "PCSK1" "FXYD1" "EGR3"

[181] "SCG5" "CGREF1" "PCP4" "GRIA2" "CALB2" "KCNK3"

[187] "GJB1" "KIF5C" "PI4KA" "CACNA2D2" "SMIM17" "MAP1A"

[193] "CNTN6" "KBTBD11" "AP3B2" "GPC5" "LYPD8" "ENC1"

[199] "PLCL1" "BCAS1" "PDE1B" "AJAP1" "SLC26A4" "LMO7"

[205] "RAB3B" "SATB2-AS1" "WASF1" "DGKZ" "AATK" "PSD3"

[211] "LINC00622" "TMEM257" "LOC283516" "STAR" "SYNGR1" "ITPR1"

[217] "CYP26A1" "PPP3CB" "COX7A1" "YWHAH" "CASKIN1" "ENTPD3"

[223] "ACVR1C" "NELL2" "CDS1" "GUCY1B3" "CPEB3" "CLDN10"

[229] "ACKR1" "MAPK8IP2" "KCNQ2" "GRP" "IL12RB2" "CHST1"

[235] "MPPED1" "NAP1L3" "PRRT3" "KLK6" "GPRASP1" "BRSK1"

[241] "FAIM2" "SPTBN2" "PVALB" "ELFN2" "PTPRN" "SLC39A12"

[247] "PLK2" "XK" "GAD2" "GFRA2" "LMO3" "GABBR1"

[253] "EXTL1" "VIPR1" "EPCAM" "RPH3A" "MAP7" "BRINP1"

[259] "RAPGEF5" "DCTN1-AS1" "INPP5F" "ZNF536" "NUAK1" "GRM1"

[265] "TF" "BASP1" "CDK5R1" "PLLP" "LDB2" "SPOCK1"

[271] "AMPH" "MATK" "EPHB6" "LINC01105" "RPRML" "PSD"

[277] "ATP2B2" "SH3GL3" "FOSB" "ASPA" "CALB1" "RTN1"

[283] "OR2L13" "GABRB1" "PAK6" "DYNC1I1" "PPFIA2" "CHGB"

[289] "NAV3" "GAD1" "MYRF" "PALM2" "SLC1A6" "LRTM2"

[295] "RAB40B" "HSPB3" "BDNF" "ANK3" "CDH9" "GABRB2"

[301] "MOG" "ST18" "FRRS1L" "HLF" "STXBP1" "LINC00632"

[307] "DNAJC6" "DUSP2" "FNDC9" "HCN1" "EEF1A2" "DMTN"

[313] "RCAN2" "SYT5" "LINC00320" "MAGEE1" "LOC401220" "CDH13"

[319] "KCNAB2" "RGS7" "ZNF365" "RIMS3" "THEMIS" "HTR2C"

[325] "LGI1" "GPR62" "KIAA0513" "DOC2A" "NEFH" "LINC00889"

[331] "CDK5R2" "PDE2A" "PTPRT" "INA" "GLS2" "UNC13C"

[337] "ARHGDIG" "TAGLN3" "RAPGEF4" "L1CAM" "LOC101929748" "SNCA"

[343] "STX1A" "NPY" "RUNDC3A" "LOC284578" "KIF21B" "KIAA0319"

[349] "CDH12" "JAZF1-AS1" "PPP1R1A" "LOC100131170" "SNAP25" "SLC26A4-AS1"

[355] "ATOH7" "HAR1A" "NELL1" "GABRA2" "HTR2A" "BSN"

[361] "CRH" "GABRA5" "FGF13" "RAB3A" "PCSK2" "CRHBP"

[367] "CDH18" "HPCA" "LINC00507" "CREG2" "RHBDL1" "MAL"

[373] "KCNK1" "ASIC2" "WNT10B" "SLC7A14" "NPTX1" "VIP"

[379] "CRYM" "RGS4" "NPY1R" "PHYHIP" "ICAM5" "CABP1"

[385] "ACTL6B" "GABRG2" "SH3GL2" "ITPKA" "SERPINI1" "SNCB"

[391] "SLC6A15" "KCNJ4" "SCN3B" "PRKCZ" "CHGA" "NRGN"

[397] "SYT1" "PDYN" "NEFM" "SYNGR3" "GRM3" "VSNL1"

[403] "SLC17A7" "TMEM235" "TAC1" "WIF1" "SNAP91" "SLC30A3"

[409] "C11orf87" "STMN2" "OLFM3" "NTSR2" "CCK" "SV2B"

[415] "CACNG3"
