# Supplementary material for: Ribonuclease A Family Member 2 Promotes the Malignant Progression of Glioma Through the PI3K/Akt Signaling Pathway
Source: Front Oncol. 2022 Jun 7;12:921083. doi: 10.3389/fonc.2022.921083 (PMC9211777; doi:10.3389/fonc.2022.921083)
Supplement: Supplementary file 4 [file DataSheet_2.pdf]

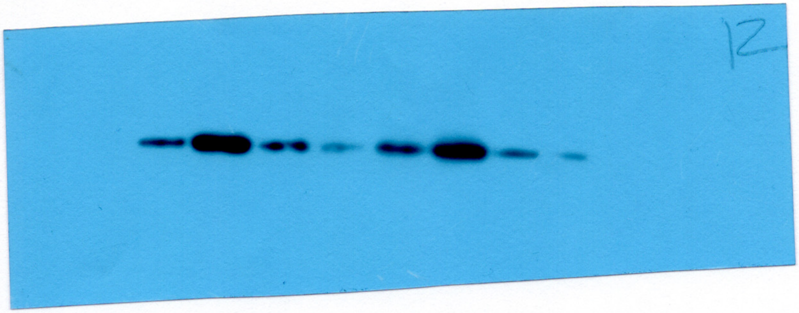
12

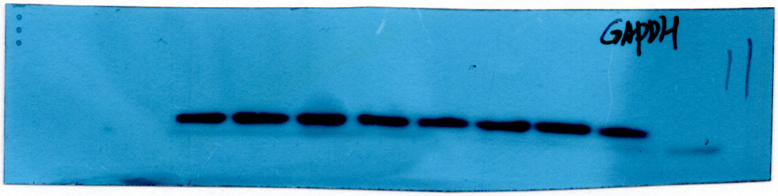
GAPDH
11

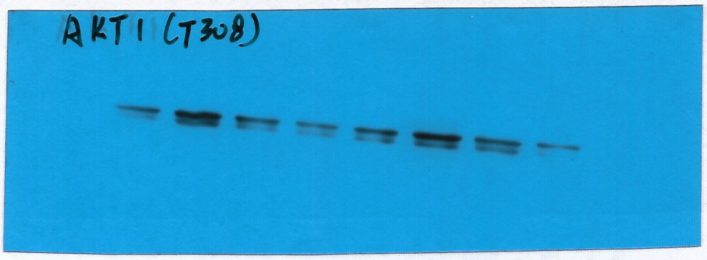
AKT1 (T308)

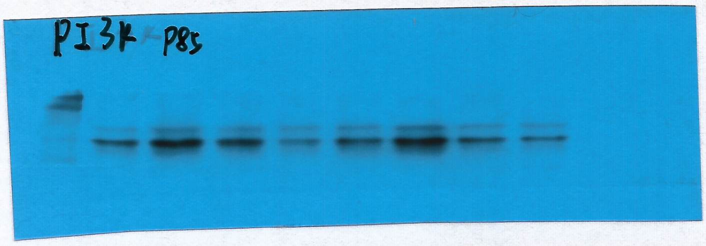
PI3K P85

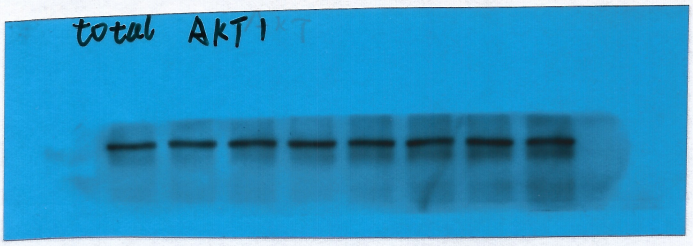
total AKT1

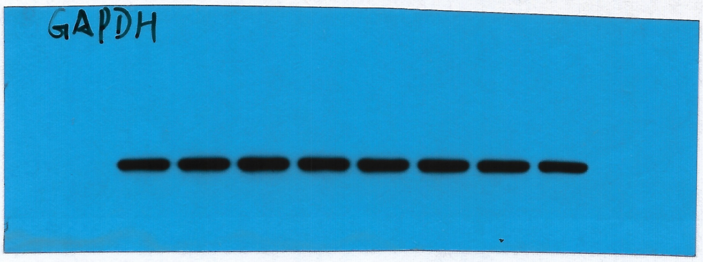
GAPDH
